# Digital image correlation as a tool for three-dimensional strain analysis in human tendon tissue

**DOI:** 10.1186/s40634-014-0007-8

**Published:** 2014-06-26

**Authors:** Thomas Luyckx, Matthias Verstraete, Karel De Roo, Wim De Waele, Johan Bellemans, Jan Victor

**Affiliations:** Department of Orthopaedic Surgery & Traumatology, University Hospitals Leuven, Weligerveld 1, Pellenberg, 3212 Belgium; Department of Orthopaedic Surgery & Traumatology, University Hospital Gent, Gent, Belgium; Department of Mechanical Construction and Production, University of Gent, Gent, Belgium; Department of Orthopaedic Surgery & Traumatology, Ziekenhuis Oost Limburg, Genk, Belgium

**Keywords:** Achilles tendon, Strain analysis, Digital image correlation

## Abstract

**Background:**

Determining the mechanical behaviour of tendon and ligamentous tissue remains challenging, as it is anisotropic, non-linear and inhomogeneous in nature.

**Methods:**

In this study, three-dimensional (3D) digital image correlation (DIC) was adopted to examine the strain distribution in the human Achilles tendon. Therefore, 6 fresh frozen human Achilles tendon specimens were mounted in a custom made rig for uni-axial loading. 3D DIC measurements of each loading position were obtained and compared to 2 linear variable differential transformers (LVDT’s).

**Results:**

3D DIC was able to calculate tendon strain in every region of all obtained images. The scatter was found to be low in all specimens and comparable to that obtained in steel applications. The accuracy of the 3D DIC measurement was higher in the centre of the specimen where scatter values around 0.03% strain were obtained. The overall scatter remained below 0.3% in all specimens. The spatial resolution of 3D DIC on human tendon tissue was found to be 0.1 mm^2^. The correlation coefficient between the 3D DIC measurements and the LVDT measurements showed an excellent linear agreement in all specimens (R^2^ = 0.99). Apart from the longitudinal strain component, an important transverse strain component was revealed in all specimens. The strain distribution of both components was of a strongly inhomogeneous nature, both within the same specimen and amongst different specimens.

**Conclusion:**

DIC proved to be a very accurate and reproducible tool for 3D strain analysis in human tendon tissue.

**Electronic supplementary material:**

The online version of this article (doi:10.1186/s40634-014-0007-8) contains supplementary material, which is available to authorized users.

## Background

Measuring the mechanical behaviour of human soft tissue remains challenging. As human soft tissue is anisotropic, non-linear and inhomogeneous in nature, its properties are difficult to characterize. Different methods have been described that are either based on contact or noncontact measurement techniques. Classically, several types of strain gauges have been used. The major downside of these measurement tools is that they are invasive in nature and act as single-point gauges, which can only record strain from one small area. Even several strain gauges cannot show regional strain and strain gradients and thus could miss critical details. Moreover, many designs only measure strain in one direction (uni-directional strain).

Image-based strain measurements on the other hand are non-invasive. Many of them optically track surface markers on the specimen during deformation to inversely calculate displacements and strain. Their resolution is mainly defined by the distance between the markers on the surface and was fairly low in many setups [1–3]. Digital image correlation (DIC) is an optical method for strain measurement that uses image recognition to analyse and compare digital images acquired from the surface of a substrate instead of surface markers [4]. By tracing a randomly applied high contrast speckle pattern using white light, displacement and strain within the specimen can be calculated from subsequent images. The initial imaging processing defines unique correlation areas known as macro-image facets, typically 5–20 pixels square, across the entire imaging area. Each facet is a measurement point that can be thought of as an extensometer point and strain rosette. These facets are tracked in each successive image with sub-pixel accuracy. Using one camera only allows for single plane measurements (2D). In this setup, out of field displacement can cause significant error. Transition to the use of 2 charge-coupled device (CCD) cameras enables three-dimensional (3D) deformation analysis of the whole area of interest and has overcome this error. As with stereopsis, using the photogrammetric principles and the 2 different images of the same object enables the calculation of the precise 3D coordinates of the entire surface. In this way, a high-resolution 3D map with strain magnitude, gradient and distribution of the entire study object can be obtained. Another major benefit is that the results from a DIC experiment are directly comparable to finite element models for model verification, iteration and boundary verification.

Despite its wide adoption in engineering research and its great potential for strain and displacement measurements in biological tissue, the reported biomechanical applications are rather limited. Some authors reported on DIC to analyse strain distribution in arterial tissue [4,5], bovine cornea [6], human tympanic membrane [7] and stapedial tendon [8], cartilage [9], composite bones [10–12] and cortical bone [13–18]. More recently, optical 2D DIC strain measurements were compared to dynamic ultrasound and ultrasound elastography measurements on animal tendon tissue, showing excellent correlation [19,20]. To our knowledge, no reports or validation of 3D DIC measurement on human tendon tissue exist.

The tensile properties of the Achilles tendon have been extensively studied both in vivo and in vitro [21–23]. Wren et al. already showed that the strain distribution in the Achilles tendon is inhomogeneous [24]. However, spatial resolution of the measurement method was low and thus local strain distribution could not be quantified. The link with clinical failure patterns remains unclear.

The first goal of this study was to determine the feasibility to evaluate the mechanical properties of the human Achilles tendon under uniaxial loading conditions with 3D Digital Image Correlation. The second goal was to compare the accuracy and reproducibility of the 3D DIC against two Linear variable differential transformers (LVDT’s).

The hypothesis for this study was that 3D DIC would be as accurate as LVDT’s in determining longitudinal strain in het human Achilles tendon and that 3D DIC would enable a full field multi-directional strain analysis of the tendon tissue.

## Methods

The research protocol was reviewed and approved by the institutional review board of the University of Ghent.

Six paired fresh frozen full limb specimens were obtained (3 left and 3 right) from 3 human donors. The mean age of the specimens was 62,3 years. The demographic variables of the specimens are described in Table 1. The specimens were store at −22°C prior to the experiment.

**Table 1:** **Demographic variables of the specimens**

**Donor nr**	**Sex**	**Age**	**Weight (kg)**
Donor A	Male	48	64
Donor B	Male	69	90
Donor C	Female	70	40

The day before the experiment the specimen was taken out of the freezer to allow 12 hours of thawing at room temperature. Next, the whole Achilles tendon was prelevated from each specimen with a calcaneal bone block. All soft tissues including the paratenon were removed from around the tendon. The calcaneal bone block was clamped between two steel plates with a rim on the end. The proximal part of the Achilles tendon was fixed in a custom made clamp with a polymer toothed rack. The tendon was kept moist at all time during the experiment using a wet cloth and water spray.

**Figure 1:**
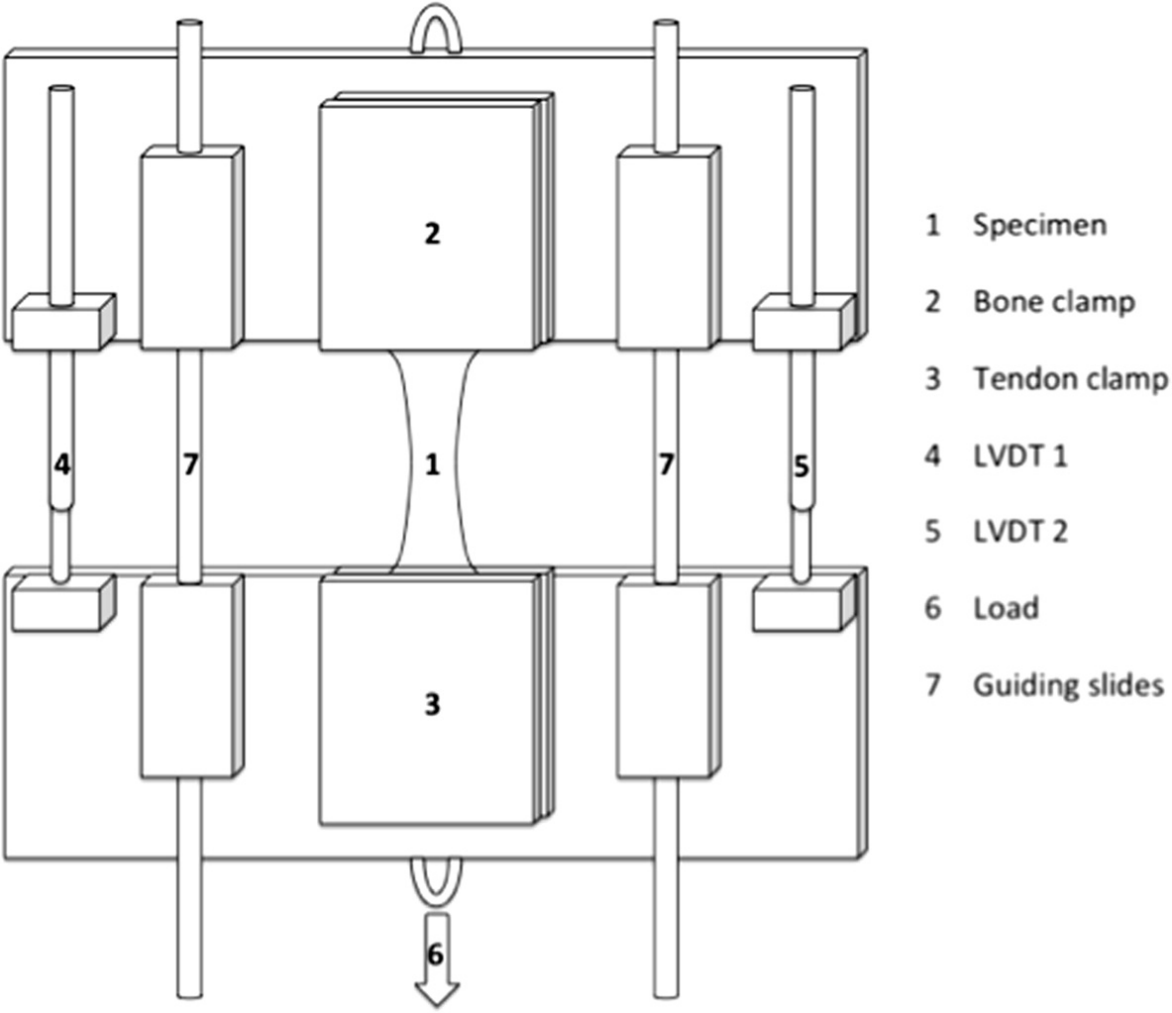
**Schematic diagram of the uniaxial loading rig.**

A custom made rig was used to apply a progressive load to the specimen (Figure 1). To prevent rotation of the specimen, the lower part of the rig moved over two slides. To assess the accuracy and reproducibility of 3D DIC, the displacement between the clamps was measured with two Linear Variable Differential Transformers (LVDTs), which measure linear displacement with an accuracy of 1 μm. The LVDTs were mounted in the frame next to the tendon. Calibrated photograph images were taken from each setup to allow accurate post-processing of the measurements.

### Specimen preparation

**Figure 2:**
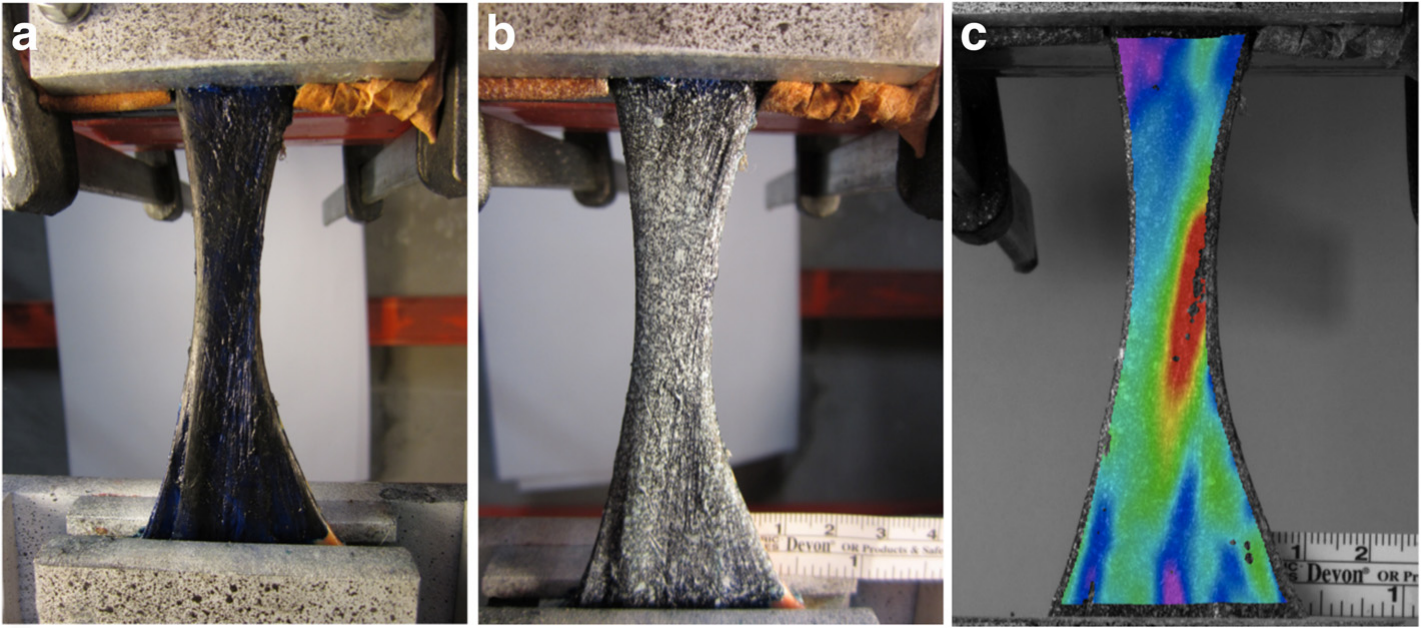
**The Achilles tendon in the clamps dyed with methylene blue (a), speckled with white paint (b) and the 3D DIC strain map plotted on the specimen showing the longitudinal strain (c).**

A modified tendon preparation technique was introduced to overcome some issues with DIC on human tendon tissue (see Discussion). The specimen was therefore first dyed with methylene blue making it appear dark blue. Methylene blue dissolved in water, penetrates the tissue colouring it in its typical dark blue colour without leaving a coat on the tissue. In this way, direct attachment of the speckle pattern onto the tissue is obtained. The dark matt background also reduced scatter. The speckle pattern is then applied with an airbrush in a matte water-based white paint instead of black paint. In this way, an optimal contrast was obtained (Figure 2).

### Digital image correlation

**Figure 3:**
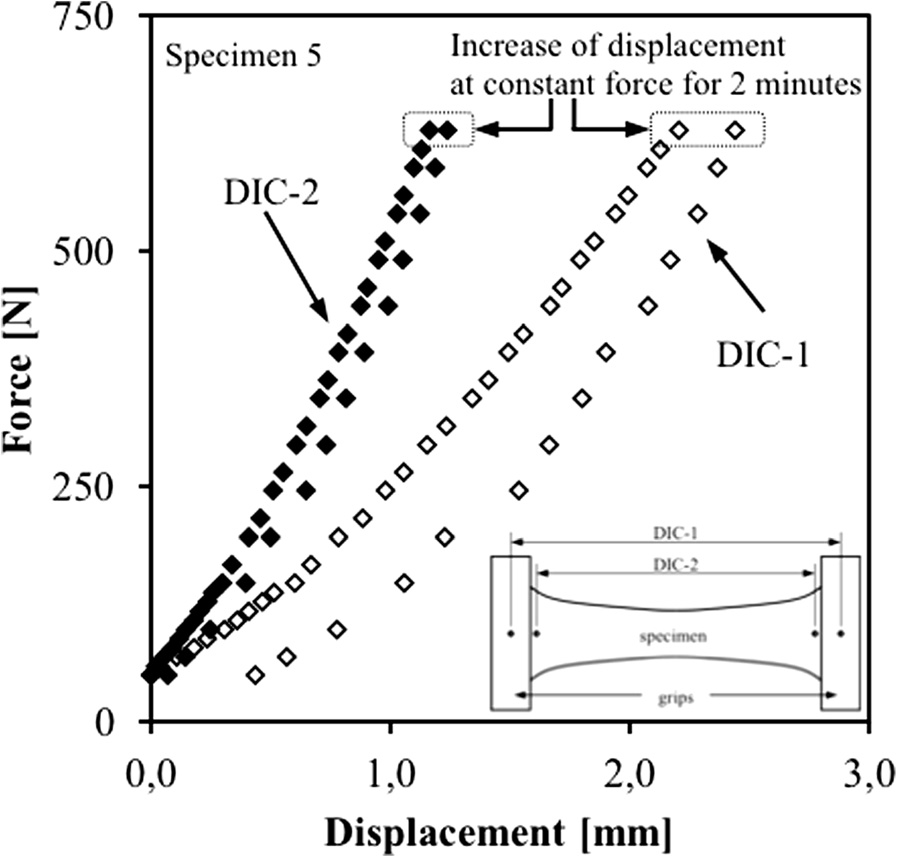
**A typical force-displacement curve of specimen 5.** Both loading and unloading phase are shown. At maximum load, the specimens were kept at constant force for roughly two minutes before unloading. For the displacements measured on the specimens (DIC-2) the difference between loading and unloading was allocated to hysteresis. For the grip-to-grip measurements (DIC-1), the difference was mainly the consequence of slip in the grips.

Two CCD cameras with a resolution of 2486 × 1985 pixels (Limess GmbH, Pforzheim, Germany) mounted on a tripod were positioned vertically in front of the loading rig and both cameras were plugged into the computer and connected to the DIC software Vic3D 2006 (Correlated Solutions, Columbia, South Carolina). A pair of halogen lamps (20 W, 150 mA, 1200 lumen, Philips Tornado) was positioned to optimize illumination and contrast of the specimen. A white screen was mounted behind the specimen for the same reason. The relative position of the cameras with respect to each other was calibrated using a high-precision 12 mm × 9 mm calibration target. Prior to the actual measurement, multiple images were captured from the preloaded condition of the specimen for an evaluation of the accuracy of the strain measurement [15]. During the test, 3 subsequent images were taken from each loading position to assess the experimental noise at the different loading conditions. The accuracy was determined in terms of scatter in the strain measurements that was measured on these images. This was done in two ways. First, the difference between the minimum and maximum strain in the overall field was evaluated. Second, the scatter in the centre-most part of the specimen was evaluated. The second method is applied to overcome disturbing effects from the boundaries of the analysed area, as it is well known that the correlation is less accurate at edges and higher strains are also observed near the site of tissue clamping [19]. The surfaces of the clamps of the rig were prepared for DIC measurement with a white basic paint and a black speckle pattern. In this way, the distance between the two clamps was recorded using the DIC as a virtual LVDT (Figure 3). For all specimens, the displacement of the grips (grip-to-grip strain), obtained through DIC (hereafter referred to as *‘DIC-1’*), was compared to the average measured displacements of the two LVDT’s fixed aside the grips. In addition to the displacement of the grips, the displacements were obtained on the specimen itself, adjacent to the grips (hereafter referred to as ‘DIC-2’). The difference between the DIC-1 and DIC-2 measurements quantified the slip in the grips.

From the obtained displacements, the corresponding strain components were derived. The longitudinal strain (ε_yy_) was defined as strain occurring in direction of the applied load. Transverse strain (ε_xx_) was defined as strain measured in a direction perpendicular to the applied load. The shear strain (ε_xy_) was defined as strain occurring in a direction angulated 45° to the longitudinal and transverse strain.

**Figure 4:**
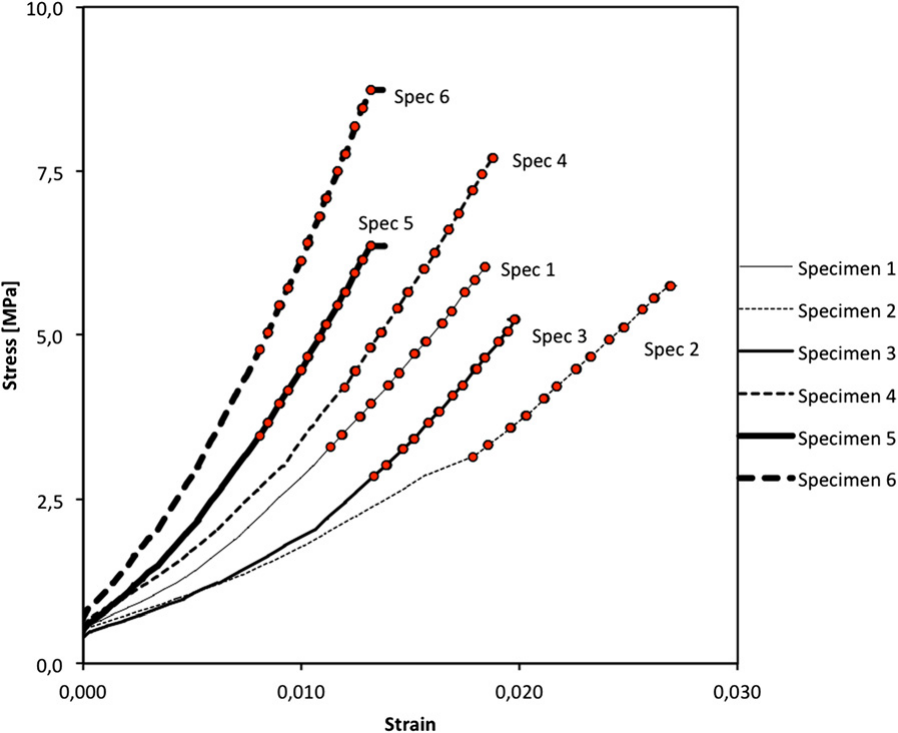
**Stress–strain curves for all 6 specimens.** The marks were placed on the linear part of the curve. The E modulus was determined from the slop of this part of the curve for each specimen. Specimen 1, 2 and 3 were not pre-loaded. Specimen 4, 5 and 6 were pre-loaded. Cfr Table 2.

To evaluate the effect of preconditioning of the tendon, a load of 607,8 N was applied during 10 minutes in specimen 4 to 6. The results for the Young’s modulus are shown in Table 2. Progressive static loading of the tendon up to 628,3 N and subsequent unloading was performed with calibrated weights. The preload was 48,9 N, additional loading in the first ten steps was done per 10 N until 100 N, from then on steps of 25 N were used until maximum load of 628,3 N. At maximum load, the specimens were kept at constant force for roughly two minutes before unloading. The stress–strain relationship is shown in Figure 4. Unloading was done in steps of 50 N. Three DIC frames of each loading position were recorded. LVDT’s attached next to the tendon continuously recorded displacement between the clamps.

**Table 2:** **Effect of pre-conditioning on the young’s modulus for each specimen**

	**Donor**	**Pre- loaded**	**Young’s modulus (MPa)**
Spec 1	Donor A Left	No	387,97
Spec 2	Donor B Left	No	294,83
Spec 3	Donor B Right	No	371,23
Spec 4	Donor C Left	Yes	514,68
Spec 5	Donor A Right	Yes	573,27
Spec 6	Donor C Right	Yes	787,68

#### Statistical analysis

For the accuracy analysis, values were obtained based on 95% confidence interval and assuming a normal distribution. A Pearson correlation test was used to evaluate the correlation between the LVDT measurements and the DIC-1 measurements. All analyses were performed using SPSS software for Mac (version 22; SPSS Inc., Chicago, Illinois).

## Results

### Accuracy analysis and comparison to LVDT measurements

**Figure 5:**
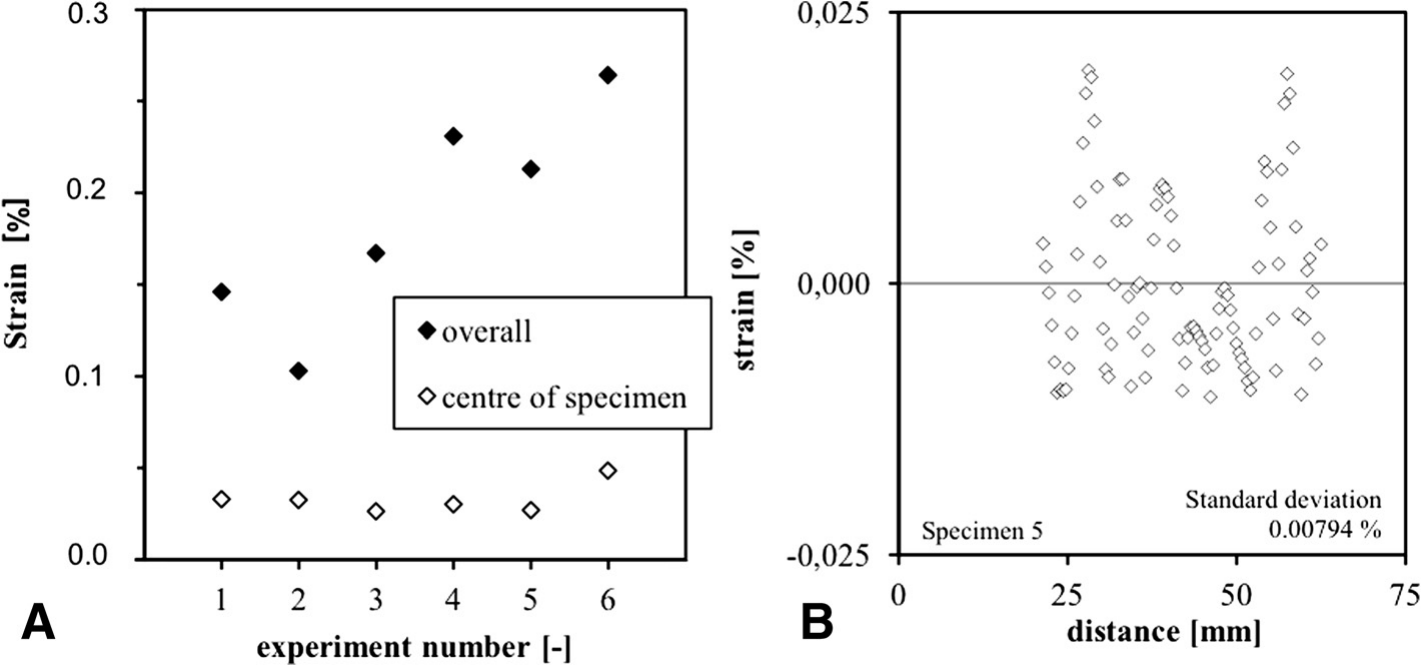
**Overview of the obtained scatter in all specimens both at the centre part and the whole tendon (A).** A typical example of central area for specimen 5 is shown in detail **(B)**.

3D DIC was able to calculate tendon strain in every region of all obtained images. The scatter was found to be low in all specimens. The accuracy of the DIC measurement was higher in the centre of the specimen where scatter values of on average 0.03% (SD 0.00794%) strain were obtained (Figure 5). The overall scatter remained below 0.3% in all specimens. The spatial resolution of 3D DIC on human tendon tissue was found to be 0.1 mm^2^.

The correlation coefficient between the 3D DIC measurements and the LVDT measurements showed an excellent linear agreement in all specimens (R^2^ = 0.99). The mean intercept and the slope of the linear correlation were 0.000 and 0.982 respectively.

**Figure 6:**
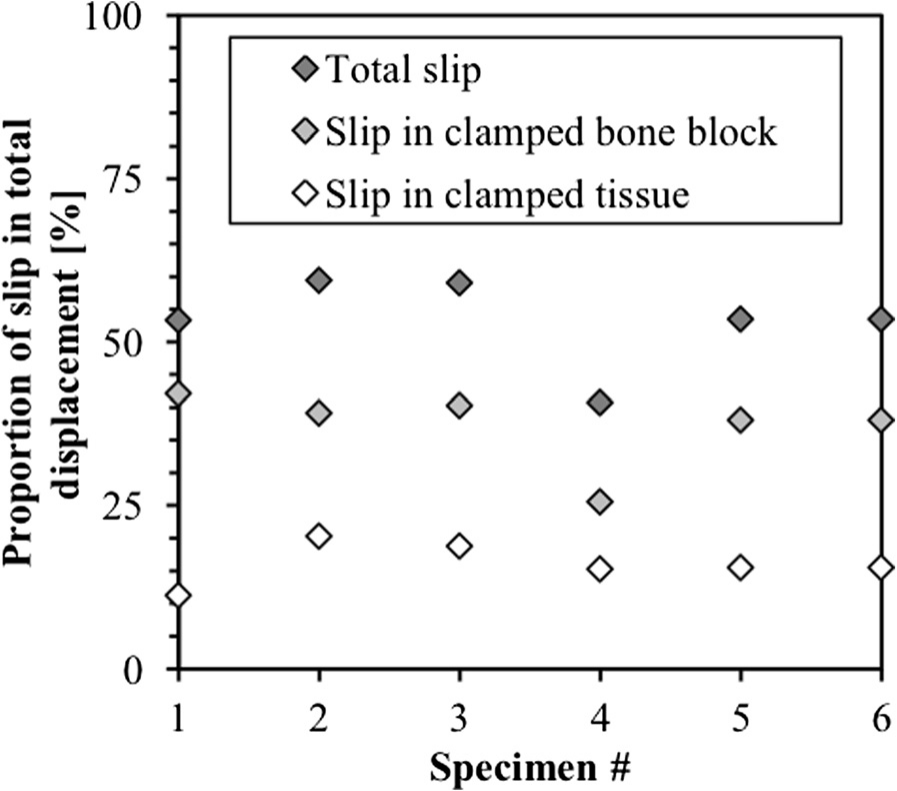
**The difference between DIC 1 and DIC 2 measurements was plotted for each specimen.** In this way, the slip in the grips was quantified. A distinction between the bone block clamp and the tissue clamp was made with the latter showing significant less slip.

Comparison of DIC-1 and DIC-2 measurements as a function of the applied load revealed a significant slip in the clamps (Figure 6). The relative error originating from not subtracting the slip in the clamps was evaluated in order to quantify the importance of the slip. Distinction is made between clamping at the tendon tissue and clamping at the bone block. The bone block clamping showed a higher slip rate. The slip at the clamps represented on average 53% of the total displacements, resulting in an overestimation of the strain in the tendon by a factor of approximately 2 if only LVDT would have been used as measurement tool. By using DIC-2 the analysis became independent of any slip.

### Strain distribution during loading cycle

**Figure 7:**
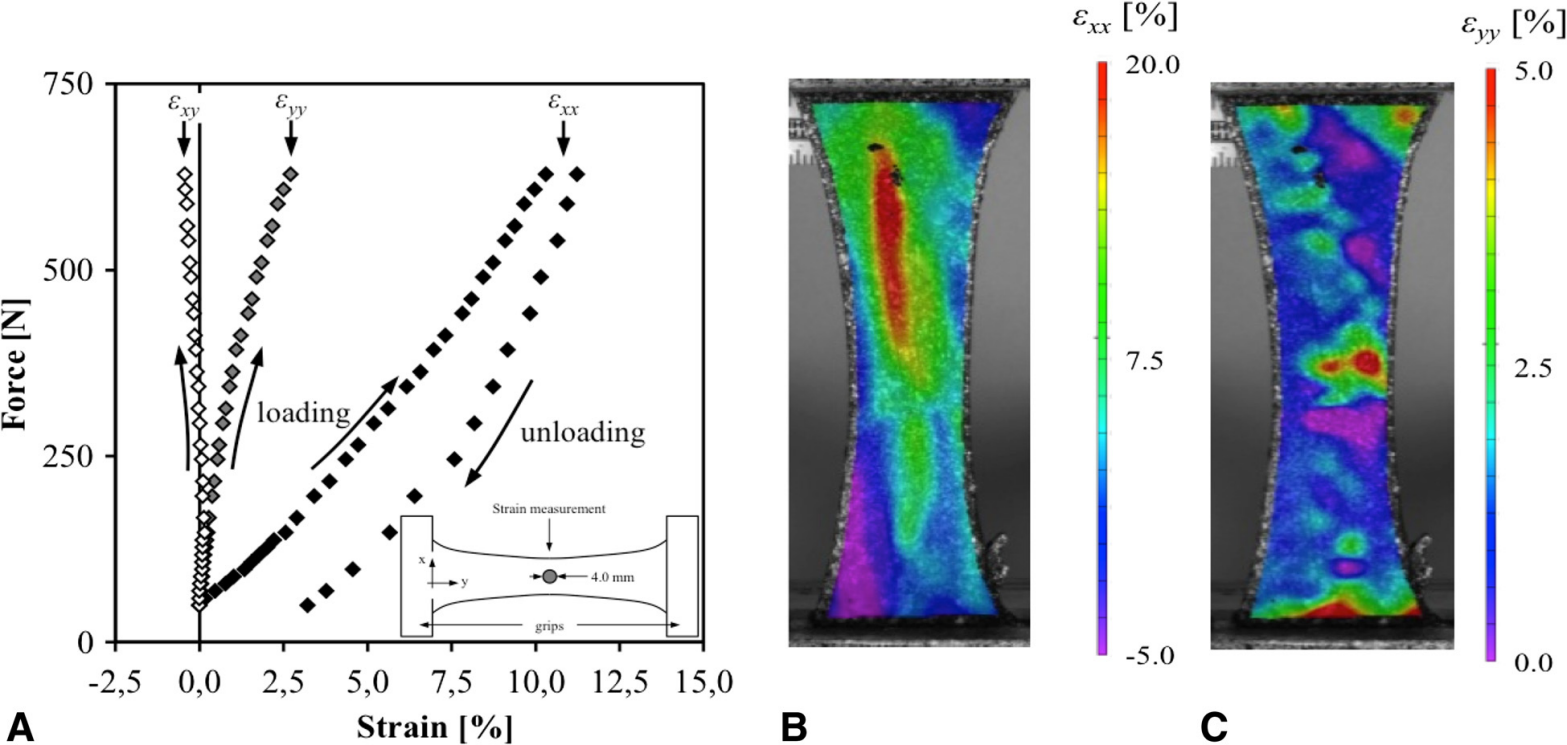
**Evolution of strain components over central area of specimen 5 as function of the applied force (A).** The shear strain ε_xy_ remained close to zero during the experiment. The corresponding strain maps at maximal load showing the transverse strains ε_xx_ **(B)** and longitudinal strains ε_yy_ **(C)** are also given.

The distribution of the average strain, measured over a circular central area with a diameter of 4.0 mm, is shown in Figure 7 as function of the applied load. Apart from longitudinal strains (ε_yy_), even larger transverse strains (ε_xx_) were observed in all specimens. The shear deformation (ε_xy_) was limited and remained close to zero during the entire test in all specimens. The presence of large positive transverse strains means that the specimen deformed (got wider) as the applied load increased. Part of the deformation was permanent (Figure 7).

#### Cumulative stain distribution

From the full field measurements, the strain can be evaluated at every tracked point of the tendon. This revealed significant regional inhomogeneity within each specimen and also between the different specimens. Although this provides in-depth insights related to the mechanical behaviour of the tendon, such approach is impractical given the strongly inhomogeneous nature of the deformation fields (Figure 7). By means of illustration, a typical DIC movie showing the loading and the unloading phase of the specimen is provided in the Additional files 1 and 2.

**Figure 8:**
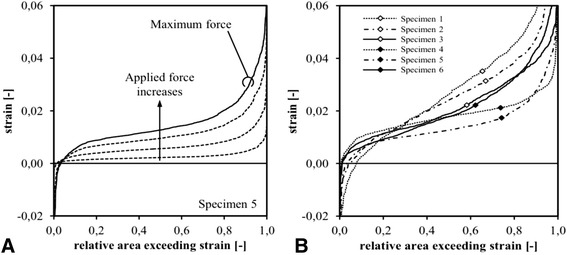
**The cumulative strain distribution plotting relative surface area of the tendon against strain. (A)** Cumulative strain distribution of specimen 5 at different loading conditions and **(B)** the cumulative strain distribution for all specimens at maximum load. The X-axis represents the relative surface area of the specimen. The Y-axis represents strain. In this way, the relative surface area showing a strain of less than and equal to a certain value is plotted. E.g. in graph **(A)** for specimen 5, at maximum load, 80% of the surface area of the tendon showed a strain of less than or equal to 2%.

To overcome the practical issues with the analysis of the data due to the local heterogeneity of the different strain maps, a cumulative strain distribution was created. To that extent, the analysed area is subdivided in squares of 1 × 1 mm and the strain is evaluated at the centre of each square. Subsequently, a cumulative distribution for the whole tendon was created for each specimen meaning that for x% of strain, the relative surface area demonstrating less then or equal to x% of strain was calculated. The cumulative distribution of specimen 5 plotted in Figure 8A showed an increasing inhomogeneity with increasing load. The cumulative distribution was obtained at maximum load for all specimens (Figure 8B), indicating that approximately 60% of the surface of each tendon has strain values between 0 and 2%. Except for specimen 1, a strain exceeding 5% (the assumed damage threshold) was only found in 10% or less of the surface area of the tendons.

## Discussion

With this study, we validated the use of 3D DIC as a highly accurate optical strain measurement tool on human tendon tissue. Despite its wide adoption in engineering research and its great potential for strain and displacement measurements in biological tissue, the reported biomedical applications are rather limited. It is our belief that the major reason for this is the technical difficulties that had to be overcome when performing 3D DIC experiments on human tissue. First of all, obtaining optimal contrast can be challenging. Most frequently, a black paint is used on a background that is light in colour. This works well on meticulously prepared fresh frozen tendon tissue, which is indeed white. However, ligaments, retinacula and fascia appear less light in colour and are difficult to examine. For specimens that are darker, the application of a matte white background is advised prior to depositing the final coating of black dots [4]. This introduces a layer between the surface of the specimen and the speckle pattern, which can cause measurement error, as the speckle pattern is no longer directly attached to the surface of the tissue. Secondly the fact that biological tissue is hydrated and has to be moist at all time of the experiment, poses some difficulties. It can be challenging to apply a speckle pattern on a moist surface. Fast-drying paint is therefore required. A moist surface also introduces scatter when the illumination is not appropriately adapted. Finally, on a moist surface, fluid drops can form during the experiment, making correlation in that region impossible. In this paper, a modified preparation technique using methylene blue was introduced. With this technique, the surface of the tendon was first dyed dark blue and then a white speckle pattern was applied. This modification, which is basically an inversion of the contrast pattern (white on black instead of black on white), can deal with most of the described problems [25]. Methylene blue is a hydrophilic molecule that is still frequently used during surgery as a method for marking soft tissue [26]. Modified tendon properties have not yet been reported with methylene blue. Moreover, in our study the methylene blue was only applied to the surface of the tendon thereby avoiding influence on the mechanical properties of the bulk of the tendon tissue. Previous techniques advocate the application of a basic layer of white paint on the study specimen before applying the black speckle pattern [4]. The major advantage of our methylene blue technique is that in our technique the speckles remain directly attached to the soft tissues as methylene blue infiltrates between the collagen fibers instead of applying an extra layer onto the surface of the tendon. It is noted that the standard deviation of the strains in the specimen is of the same order of magnitude as in experiments on the steel blocks. Thus, it is concluded that the accuracy on tendon tissue was very high. This adaption might expand the research applications of 3D DIC to other human collagenous tissues like ligaments and retinacula that were otherwise difficult to examine because of the lack of contrast [27].

Some authors reported on DIC to analyse strain distribution in arterial tissue [4,5], bovine cornea [6], cartilage [9], composite bones [10–12] and and cortical bone [13–18]. In most of these reports, a 2D technique is used. To our knowledge, this is the first validation report of 3D DIC measurement on human tendon tissue. The major advantage of 3D DIC over the 2D analysis is the fact that rigid body motions can be calculated from the original pixel registration and thus can be subtracted. This opens perspective for strain measurement on a moving object (e.g. knee squat) [27]. The 2 CCD camera setup allows the calculation of a detailed 3D surface coordinate map. Stain and strain gradients can be plotted on this map.

3D DIC enabled visualisation of multidirectional strain components that would have been missed using classic strain gauges. In our experiment for example, a significant amount of strain was observed in a direction perpendicular to the applied load (Figure 7). This transverse strain exceeded the longitudinal strain component. Although it appears counterintuitive, this was expected, as the specimens were slightly convex. Accordingly, the convexity decreases when loaded in longitudinal tension. The specimens got straighter and thus wider, mainly in their central portion. All previous Achilles tendon loading experiments were not able to capture this transverse strain component. It might in fact have an influence on the observed tendon rupture patterns. Also shear strain could be quantified from our data.

Due to its non-contact nature, the 3D DIC was able to quantify and subtract the slip that is inevitably seen at the tendon-interface. In this way, our analysis became independent of any slip at the grips. Failure to subtract this slip might be part of the explanation for the large variance in failure stresses (38–86 MPa) and failure strains (7,5 – 16,1%) for the Achilles tendon reported in literature [21,24,28].

As 3D DIC provided a high-resolution full field analysis, data of the whole tendon at sub-pixel level were provided. This enabled us to visualise the regional inhomogeneity in strain distribution that is typical for biological tissue. This regional inhomogeneity stresses the importance of doing such a full field analysis. Classic strain gauges are not able to provide these data unless an infinite number of them would be used. This is in fact how the 3D DIC technique should be looked at. It was shown that regional strain could be quite different from the overall strain in a specimen. To capture all the data provided by the strain map, the cumulative strain distribution was introduced (Figure 8). In contrast to Young’s modulus, this tool is independent of the volume of the clamped tendon and might therefore shed a new light over failure patterns in tendon tissue. It is frequently observed in strain experiments that maximum mid-substance strains are smaller than the grip-to-grip strain [19]. This phenomenon is well documented in the literature [29], and is likely the result of higher strains arising near the site of tissue clamping and slip at the grip-tendon interface. Therefore, instead of defining tendon failure by a certain grip-to-grip strain during a loading experiment (e.g. 10%), it could be more appropriate to state that tendon failure occurred when a certain percentage its surface area (e.g. 30%) reached a certain threshold of strain (e.g. 8%). This cumulative failure strain will in fact be lower then the corresponding grip-to-grip strain due to the regional inhomogeneity in strain distribution. As relative surface area (%) is used in this measure, it becomes independent of tendon volume. This cumulative distribution can thus provide important insight in the displacement energy absorption within the tendon and might show better correlation with damage accumulation and failure patterns. Part of our further research will therefore focus on the validation of the cumulative strain distribution and the determination of the cumulative damage and failure thresholds in different tendon tissues.

One of the downsides of this study is the rather limited sample size of six specimens. Nevertheless, the correlation between the measurement methods was found to be linear and strong. Adding more specimens would therefore not change the statistical conclusion. Another downside of the DIC technique is that its analysis is limited to the properties of the superficial layer of a tissue sample. However, the potential of using the surface measurement of 3D DIC to assess mechanical states throughout the bulk of a tissue has been suggested [30]. Moerman et al. showed that the use of 3D DIC in combination with inverse finite element analysis is a valuable tool to non-invasively determine the bulk material properties of soft tissue [31]. The use of Methylene blue might also influence the tendon properties. However, this was not reported so far. We minimised a potential effect by only applying methylene blue to the surface of the tendon. In this way, an influence on the mechanical properties of the bulk of the tendon tissue was avoided. The fact that a contrast pattern has to be applied, limits the research possibilities of the technique mainly to ex-vivo experiments. Recent research has focused on the possibilities of the digital image correlation technology to track the texture of ultrasound images. As ultrasound is frequently used to image musculoskeletal tissue, this technique allows in vivo strain measurement. Several authors showed an excellent correlation between classic 2D DIC measurements and 2D ultrasound elastography [19,20,32].

## Conclusion

3D DIC proved to be a very accurate and reproducible tool for strain measurement in human tendon tissue. The introduction of a high-resolution full field strain analysis might shed new light over previous insights in damage accumulation and failure patterns of tendon tissue. Further research will be directed to these topics.

## Additional files

Additional file 1:
**A typical example of a strain movie file showing the longitudinal strain component plotted on specimen 3 during loading and subsequent unloading.** The highest strains are observed at the centre of the specimen and in the regions close to the grips. Additional file 2:
**A typical example of a 3D strain plot of specimen 5. The longitudinal strain component is plotted on the specimen.** The highest strains are observed at the centre of the specimen and in the regions close to the grips. A part form longitudinal deformation, also a displacement in the Z-plane is observed. Failure to quantify this out of plane movement can induce measurement error. The use of 3D DIC clearly demonstrates its advantages over 2D DIC measurements at this point.
